# Primary neuroendocrine tumors of the thymus: Clinical review of 22 cases

**DOI:** 10.3892/ol.2014.2490

**Published:** 2014-09-01

**Authors:** ZHENGBO SONG, YIPING ZHANG

**Affiliations:** 1 Department of Chemotherapy, Zhejiang Cancer Hospital, Hangzhou, Zhejiang 310022, P.R. China; 2 Key Laboratory Diagnosis and Treatment Technology on Thoracic Oncology, Hangzhou, Zhejiang 310022, P.R. China

**Keywords:** thymus, neuroendocrine tumor, prognosis, treatment

## Abstract

Primary neuroendocrine tumors of the thymus are rare mediastinum tumors, which present a distinct type of tumor, which exhibit morpholgical and biological neuroendorcine features including the production of numerous biogenic amines. The aim of the present study was to evaluate factors influencing long-term survival in patients with primary neuroendocrine tumors of the thymus. A total of 22 patients exhibiting primary thymic neuroendocrine tumors, who were treated at the Zhejiang Cancer Hospital (Hangzhou, China), between 1995 and 2012 were reviewed. Survival curves were plotted using the Kaplan-Meier method and the Cox proportional hazards model was used for multivariate analysis. The overall five-year survival rate was 45.5% and the median survival time was 59 months in all of the patients. Histological grade (P<0.001), Masaoka-Koga stage (P=0.003) and surgical resection status (P=0.004) were identified to be associated with patient survival time. Furthermore, multivariate analysis identified that the histological grade was an independent prognostic factor, which was applicable to all patients (P=0.009). Therefore, the histological grade and Masaoka-Koga stage, as well as surgical resection status present three prognostic factors in patients exhibiting primary thymic neuroendocrine tumors.

## Introduction

Neuroendocrine tumors of the thymus are rare, with an annual incidence of 0.01/100,000 in the USA (1). The histogenesis of neuroendocrine tumors varies and the tumor may arise from ectopic tissues in the mediastinum or present within the thymus (2). Thus, the histopathological classification, prognosis and treatment of primary neuroendocrine carcinomas of the thymus remain controversial.

According to the World Health Organization (WHO) (2), neuroendocrine tumors are included in the thymic carcinoma group and classified as two histopathological types; well-differentiated neuroendocrine carcinomas (typical and atypical carcinoid) and poorly differentiated neuroendocrine carcinomas (small cell carcinoma and large cell neuroendocrine carcinoma). The well-differentiated neuroendocrine carcinomas show a low grade of biological aggressiveness, while poorly differentiated neuroendocrine carcinomas are considered to be high-grade neuroendocrine tumors. As there have only been a small number of patients with neuroendocrine tumors of the thymus reported in the literature (3–7), a consensus has not been reached concerning the prognostic factors of primary neuroendocrine tumors of the thymus.

The aim of the present study was to evaluate the factors influencing long-term survival in 22 patients with primary neuroendocrine tumors of the thymus and to explore the role of various prognostic factors.

## Patients and methods

### Patient eligibility

The records of 22 patients exhibiting primary neuroendocrine tumors of the thymus, who were treated at the Zhejiang Cancer Hospital (Hangzhou, China), between 1995 and 2012, were reviewed. The 22 patients included 14 males and eight females, with a median age of 49.5 years. The histological type was determined according to the 2004 WHO classification (2) and the staging was performed for all patients according to the Masaoka-Koga system (8). Recurrence or metastases were identified using chest computed tomography (CT), as well as ultrasound and/or CT of the abdomen. The study was approved by the ethics committee of Zhejiang Cancer Hospital (Hangzhou, China).

### Patient treatment

A total of ten patients underwent surgical resection following first diagnosis. A total of 9 patients received chemotherapy, 8 patients received radiation therapy, 3 patients received chemotherapy and radiotherapy and two patients received no futher treatment. The detailed treatment of the 22 patients is shown in Table I.

### Follow-up

Patients were followed up every three to six months for the first five years, and once per year thereafter. Each patient’s medical history, details of physical examinations and thoracic CT scans were recorded. The last follow-up was on Jan 30, 2013, with a median follow-up period for all patients of 109 months (range, 15–185 months).

### Statistical analysis

Survival curves were calculated (using the Kaplan-Meier method) commencing from the date of the confirmed pathology to the date of mortality or the last follow-up. The log-rank test was used to compare overall survival (OS) time between different factors, including gender, age, tumor stage and surgery status. Multivariate analysis was performed using the Cox proportional hazards model and statistical analysis was performed using the SPSS version 15 software (SPSS, Inc., Chicago, IL, USA). Confidence intervals were calculated at the 95% level and P<0.05 was considered to indicate a statistically significant difference.

## Results

### Clinical characteristics

The clinical characteristics of the 22 patients are listed in Table I. The 22 patients enrolled in the present study included 14 males and eight females, with a median age of 49.5 years. In total, 10 of the 22 individuals underwent surgery. The pathological stage was I and II in nine patients, and III and IV in 13 patients. According to the WHO criteria (2), based on the histopathological differentiation, all 22 cases were divided into two types; well-differentiated (n=13) and poorly differentiated (n=9) neuroendocrine carcinomas.

### Survival analyses

**Figure 1:**
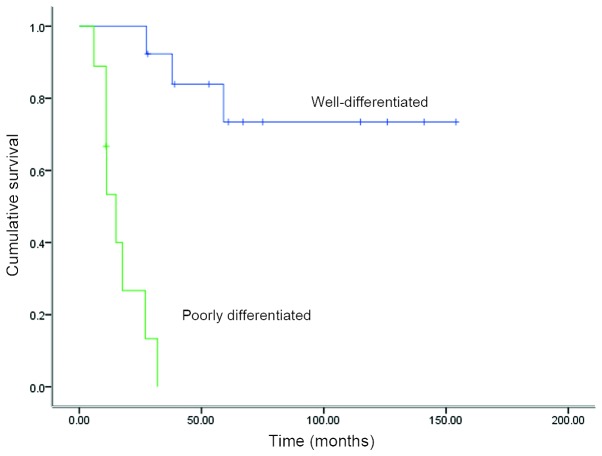
Kaplan-Meier curves comparing the survival times of patients with well- and poorly differentiated neuroendocrine carcinomas (P<0.001).

**Figure 2:**
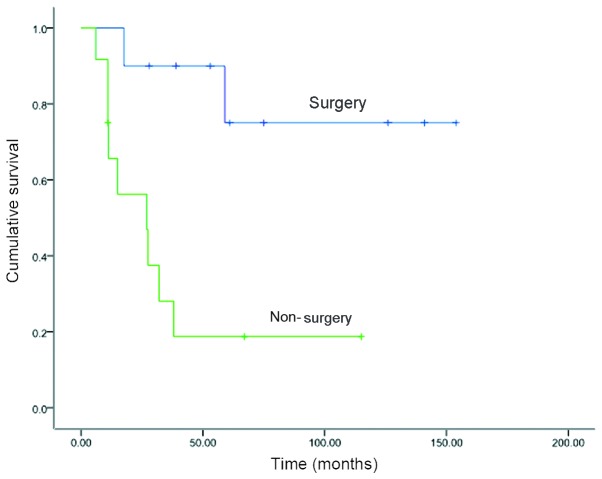
Kaplan-Meier curves comparing the survival time of patients that underwent surgery with those that did not (P=0.004).

**Figure 3:**
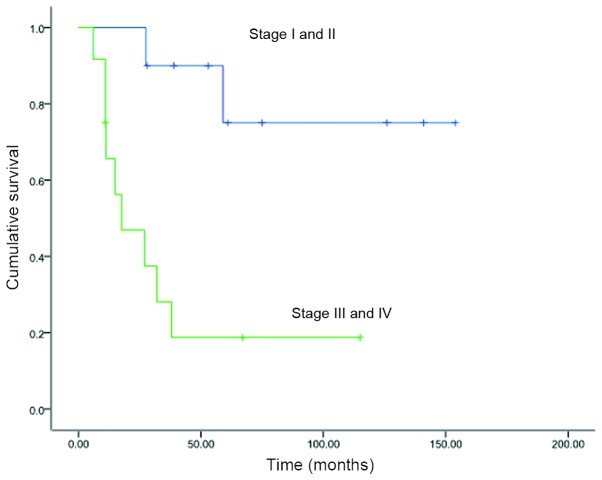
Kaplan-Meier curves comparing the survival time of patients with early (Stage I–II) and late (Stage III–IV) stage tumors (P=0.003).

Table II shows the results of the univariate analyses of the clinicopathological factors evaluated in the present study. At present, a total of 11 patients have survived, however, 11 patients succumbed to the disease prior to the final follow up date. The median survival time for all patients was 59 months, and the five-year OS rate was 45.5%, with ten patients surviving longer than five years. Patients with poorly differentiated neuroendocrine carcinomas, that were stage III and IV, and did not undergo surgery exhibited a significant correlation with poor OS (Table II and Figs. 1–3).

A multivariate Cox proportional hazards model was constructed, which accounted for age, gender, tumor size, histological grade and surgery as variable factors. The histological grade was the only independent prognostic factor identified for OS (P=0.009; Table III).

## Discussion

Primary neuroendocrine tumors of the thymus are rare, with ~400 cases reported in the literature to date; the majority of which are case reports (3–7, 9–11). The median age at diagnosis has been relatively young in the majority of studies, ranging between 40 and 60 years. A male predominance has also been observed in the literature, which is consistent with the findings of the current report.

In a series of 15 patients reported by Fukai *et al* (5), the five-year survival rate was 33%, and of the 14 cases reported by de Montpreville *et al* (3) the five-year survival rate was 31%. The overall five-year survival rate in the present study was 45.5% (Table II), which is consistent with that reported in earlier studies (3,5). However, the median OS was shorter than that of previous reports (1,12,13), which may be due to more than half of the patients reported in the present study not undergoing surgery.

As the diagnosis of primary neuroendocrine tumors of the thymus is rare, only a small number of retrospective studies are available. Therefore, a standard therapeutic strategy has not yet been defined. Surgery remains the standard method for the treatment of thymic tumors compared with non-surgical options according to the Surveillance, Epidemiology, and End Results database analysis (1). In the present study, a significant difference in the OS of patients was identified between those who underwent surgery and those who did not. However, the prognostic factors currently in use for primary neuroendocrine tumors of the thymus, including histological grade, Masaoka-Koga grade and surgery status, remain controversial. To date, the histological grade, Masaoka-Koga stage and surgical resection status have been validated as prognostic factors. In addition, in the present study, carcinoids showed the optimum prognosis, while large cell neuroendocrine carcinoma and small cell carcinoma were associated with a poor prognosis (Fig. 1). Furthermore, patients with Masaoka-Koga stages III and IV showed a poorer prognosis than stage I and II patients (Table II and Fig. 3).

The major limitations of the current study were its retrospective nature and the subjects being obtained from a single institution. In addition, a small level of heterogeneity was identified among the surgical and non-surgical patients, which may have influenced the analysis of the prognosis. However, despite the small patient population that was used in this retrospective study, the results are considered to be meaningful.

In conclusion, thymic neuroendocrine tumors are associated with a discriminative prognosis. The histological grade, Masaoka-Koga stage and surgical resection status were identified to be prognostic factors. However, further study is required to fully validate the prognostic factors and determine a standard treatment for thymic neuroendocrine tumors.

## Figures and Tables

**Table I:** Characteristics of 22 patients.

Case	Gender/ age, years	Histology	Masaoka-Koga stage	Surgery	Treatment	Metastasis		OS, months

						At diagnosis	During disease	
1	M/70	AC	I	Yes	No	No	No	154+
2	M/50	SCC	IV	No	Chemo	Supraclavicular LN	Lung	11.2
3	F/39	LCNEC	III	Yes	Radiotherapy	No	Lung, bone	17.6
4	M/50	AC	IV	No	Chemo	No	Bone	27.5
5	F/49	TC	II	Yes	Radiotherapy	No	No	126+
6	F/40	AC	II	Yes	Radiotherapy	No	No	141+
7	M/38	AC	II	Yes	Radiotherapy + Chemo	No	No	75+
8	F/51	AC	II	Yes	Radiotherapy + Chemo	No	No	61+
9	F/52	AC	II	Yes	Radiotherapy	No	No	53+
10	M/29	AC	II	Yes	Radiotherapy	No	No	28+
11	M/29	AC	I	Yes	No	No	No	39+
12	M/51	SCC	III	No	Radiotherapy + Chemo	No	Liver, lung	27
13	M/24	TC	II	Yes	Radiotherapy	No	Liver, bone	59
14	M/57	AC	III	No	Radiotherapy	No	No	67+
15	M/61	LCNEC	IV	No	Chemo	Lung	Bone	11+
16	F/48	SCC	IV	No	Chemo	Supraclavicular LN	Lung	11
17	F/43	LCNEC	III	No	Radiotherapy	No	Bone, lung	32
18	M/59	SCC	IV	No	Chemo	Liver	Supraclavicular LN	6
19	M/55	LCNEC	IV	No	Chemo	Lung	Bone	11
20	M/55	SCC	IV	No	Chemo	Bone	Lung	15
21	M/49	AC	IV	No	Chemo	Lung	Liver	38
22	F/47	TC	IV	No	Chemo	Lung	LN	115+

M, male; F, female; AC, atypical carcinoid; LCNEC, large cell neuroendocrine carcinoma; SCC, small cell carcinoma; TC, typical carcinoid; Chemo, chemotherapy; LN, lymph node; OS, overall survival.

**Table II:** Univariate analysis of patient OS rate.

Variable	Five-year OS rate, %	P-value
Gender		0.311
Male	34.1	
Female	62.5	
Age, years		0.357
≥50	40.9	
<50	49.1	
Tumor size, cm		0.351
>5	40.1	
≤5	50.0	
Grade		<0.001
Poorly differentiated	73.4	
Well-differentiated	0.00	
Masaoka-Koga stage		0.003
I+II	75.0	
III+IV	18.8	
Surgery		0.004
Yes	75.0	
No	18.8	

OS, overall survival.

**Table III:** Multivariate analysis of patient overall survival rate.

	Overall survival		
	
Variable	HR	95% CI	P-value
Gender (male vs. female )	3.977	0.699–22.610	0.120
Age, years (≥50 vs. <50)	0.968	0.880–1.065	0.501
Tumor size, cm (>5 vs. ≤5)	3.466	0.651–18.457	0.145
Grade (poorly differentiated vs. well-differentiated)	51.074	2.698–966.717	0.009
Masaoka-Koga stage (III+IV vs. I+II)	1.824	0.027–123.576	0.780
Surgery (yes vs. no)	0.243	0.005–11.111	0.469

HR, hazards ratio; CI, confidence interval.
